# An International Aircraft Transport of a Neonate From Georgia to Japan

**DOI:** 10.7759/cureus.28952

**Published:** 2022-09-08

**Authors:** Soichiro Saeki, Hidetoshi Taniguchi, Hitomi Arahori, Yasuji Kitabatake, Keiichi Ozono

**Affiliations:** 1 Epidemiology and Public Health, Center Hospital, National Center for Global Health and Medicine, Tokyo, JPN; 2 Medicine, Osaka University, Osaka, JPN; 3 Pediatrics, Osaka University, Osaka, JPN

**Keywords:** referral and consultation, migrant health, international long-distance transport, healthcare insurance, neonate

## Abstract

International air transport over long distances necessitates considerable effort. It is even more challenging when the patient is a neonate and has a congenital disease. We hereby report a case of an international aircraft transport of a neonate from Tbilisi, Georgia to Osaka, Japan. The patient was transported to Osaka University Hospital after being diagnosed with a double outlet right ventricle (DORV), requiring surgical intervention. This unique experience has raised four issues: 1) language issues for referral and consultation; 2) medical equipment and healthcare professionals required to accompany the transport for adequate care; 3) scheduling of the international flight; and 4) the administrative procedures such as birth certificate, passport, and healthcare insurance. In this report, we describe how the patient was successfully transported, received treatment, and discharged home.

## Introduction

Emergency international air transport of neonates may occur when unexpected preterm delivery has taken place or undiagnosed congenital anomalies are found in an infant when advanced neonatal care is not available in an isolated place. There are a significant number of case reports of neonatal transport by aircraft but international long-distance transport of neonates using medically equipped aircraft is extremely rare. It is largely because most such infants receive advanced neonatal care locally, and if such care is unavailable, they may not survive until the transport team reaches them. Other possible barriers to long-distance air transport include hesitation in accepting medical staff and the overall perceived burden on the families. A previous case report highlighted the clinical and planning difficulties in long-distance neonatal transport from China to Italy [1]. In addition to medical conditions, social criteria such as the departing/accepting countries, the nationality of the patients and their families, and health insurance coverage have the possibility of turning into factors that could affect the medical care the patients receive. As a result, it is of significant importance to accumulate knowledge and experience of key findings of cases with different medical and social backgrounds. Therefore, we hereby report a case of an international aircraft transport of a neonate from Tbilisi, Georgia to Osaka, Japan.

## Case presentation

A dizygotic male infant was born in Tbilisi, Georgia at 36 weeks gestational age by an emergency cesarean section due to maternal hypertensive disorder in pregnancy. His mother is Japanese while his father is from the United States. This was a birth given by a Georgian surrogate mother. He was small for gestational age with a birth weight of only 1200g. He had tachypnea and the heart murmur was heard. The infant was diagnosed with a double outlet right ventricle (DORV), but the parents were informed that no surgeon in Georgia could perform heart surgery on an infant with his body weight. Therefore, the parents contacted the department of cardiovascular surgery at Osaka University Graduate School of Medicine. The physicians in Georgia were unable to communicate in English, and their referral letter was written in Georgian. Therefore, the surgeon at Osaka University Hospital had to obtain medical information mostly from the parents via e-mail and web conference services. The parents requested transfer by a private international transfer agency (Pediatric Air Ambulance, Munich, Germany).

**Figure 1:**
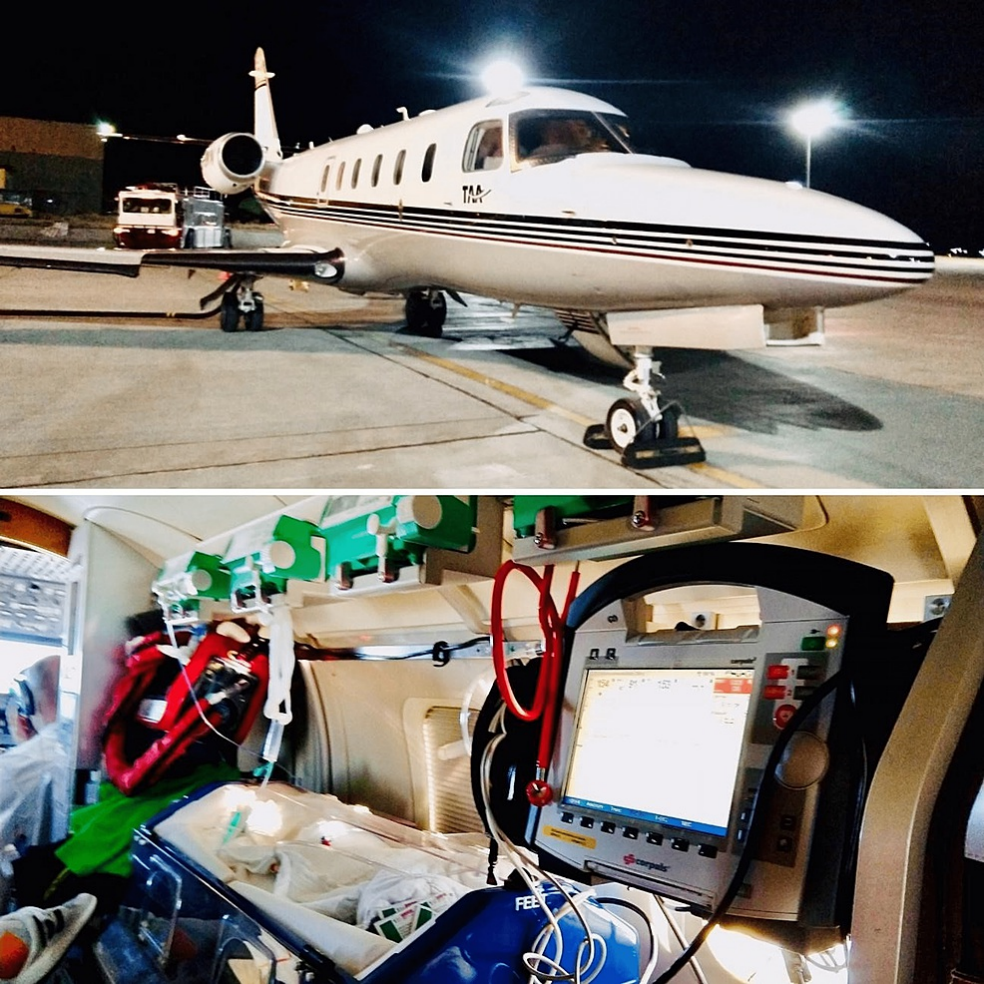
Aircraft and equipment used for the transfer. Picture courtesy, the patient’s parents.

A pediatric cardiologist and a nurse from the transferring company departed Munich to Tbilisi and then transferred the patient as well as his family to Osaka using an aircraft. The aircraft departed Tbilisi International Airport (TBS), and arrived at Kansai International Airport (KIX) at early dawn, with brief stops for fuel in Tibet and Ulaanbaatar. Although planning was discussed via the Internet, due to complications with the routing of the transfer with the authorities in the People’s Republic of China and the Russian Federation, we were not notified of the arrival date until three days prior to arrival. At the time of departure from Georgia, the medical team had not provided treatment such as oxygen, mechanical ventilation, or injection of diuretics. A peripherally inserted central venous catheter (PICC) was placed and infusion had been made along the way. The aircraft was equipped with the Baby Pod II® incubator (Advanced Healthcare Technology Ltd., Suffolk, UK); a mechanical ventilation device (Hamilton T1®, Hamilton Medical, Bonaduz, CH); a blood gas analysis device (Alere EPOC®, Siemens Healthineers, Erlangen, DE); a monitor equipped with an electrocardiogram (ECG), defibrillator, pacer; saturation of peripheral oxygen (SpO2), non-invasive blood pressure (NIBP) and invasive blood pressure (IBP) sensors (Corpuls C3®, Corpuls, Bayern, DE); and syringe pumps (Perfusor®, B. Braun Medical Inc., Melsungen, DE). These enabled the medical team to provide the best monitoring and care during transport (Figure 1).

The patient arrived at Osaka University Hospital on postnatal day (PND) 20 via commercial ambulance from the airport. The previously diagnosed DORV was confirmed by echocardiography. Chest radiograph showed an increase in pulmonary blood flow and diuretics were initiated. Physical examination revealed increased head circumference and hepatomegaly. Computed tomography of the head and abdomen revealed no ventriculomegaly, no brain tumor, and no space-occupying lesion in the liver. He had a normal karyotype (46, XY). The blood test was significant for high brain natriuretic peptide (553.9 pg/dL). Parenteral nutrition with amino acids and lipids was given from PND 22 to PND 30. On PND 31, pulmonary artery banding (PAB) was performed. After confirming no abnormalities in the brain by MRI on PND 77, the patient was discharged home on PND 81. Meanwhile, the patient’s mother applied for residency and public health insurance in Osaka, Japan, which enabled the patient to stay in Japan while their medical expenses were covered as the dependent of his mother.

## Discussion

In this case, there were difficulties and problems generally seen in any international air transport as well as neonatal international transport. The obstacles highlighted in this case can be grouped into four categories: 1) language issues for referral and consultation; 2) medical equipment and healthcare professionals required to accompany the transport for adequate care; 3) scheduling of the international flight; and 4) the administrative procedures such as applying for a birth certificate, passport, and healthcare insurance.

Firstly, linguistic issues hindered communication among the medical teams. Information provided by the former physician was in Georgian. Although Japan has been introducing medical interpreters as a nation to deal with patients with limited-Japanese proficiency [2], according to the Georgian Embassy in Japan, as of March 2021, official Georgian language assistance can only be provided by the embassy, and no commercial professional service is currently available.

Secondly, a neonate who needs to be transported overseas may face unique health situations. The potential risk of aircraft transport might have decreased partial pressure of oxygen as this infant had congenital heart disease [3]. However, this effect might have been negated as he had high blood flow to the pulmonary arteries. Another risk might be apnea of prematurity [4], which can be managed by the medical team on board. In addition, although modern neonatal aircraft transfer has been known to be relatively safe [5], the unstable environment in the aircraft such as high altitudes and vibration could negatively impact a neonate’s health [6]. Therefore, a medically equipped aircraft and healthcare professionals are required on board, which can make the transfer fees quite expensive for the family [7].

Thirdly, there was a difficulty in routing management. Our case encountered complications with the routing of the transfer with the authorities in the People’s Republic of China and the Russian Federation. As a result, the schedule was fixed on a Friday, and the patient arrived before dawn the following Monday.

Fourthly, social issues may arise in the care of neonates arriving from abroad. As newborns are required to obtain a birth certificate and passport at the same time, this complicated documentation arrangement may hinder the patient from being able to leave the foreign country smoothly. When neonates born abroad are to return to Japan, a passport will not be issued until a certificate of family records has been obtained. This may be waived in emergencies, but registration of a birth certificate is still required at the embassy to obtain a single-use emergency travel document. In this case, the patient’s father’s nationality qualified the patient to obtain citizenship in the United States, therefore obtaining an American passport for international departure was fairly easy. Furthermore, for foreign patients, the problem of expensive medical costs arises in Japan [8]. In this case, the patient was able to obtain public medical insurance by becoming the mother’s dependent, who had a residency in Japan.

## Conclusions

To summarize, international transfers of neonates can face many difficulties medically, linguistically, financially, and juristically. Medical institutions must be aware of such possibilities, and provide adequate preparations by organizing resources to address international situations such as linguistic assistance which may not be provided by medical interpretation companies, and financial support for neonatal transports.
